# Harvard Dental Alumni Association

**Published:** 1902-01

**Authors:** 


### HARVARD DENTAL ALUMNI ASSOCIATION.

MEETING called to order by President Cecil P. Wilson. Dr. Thomas Fillebrown, of Boston, Mass., presented two cases of cleft palate.

*Dr. Fillebrown.*—This first patient is a child two years old, born with a wide cleft in the palate, and harelip. At seven weeks old the fissure in the hard palate was closed. Two weeks later the lip was operated on; after that the soft palate was united. At twelve weeks of age a slight secondary operation was done on the lip, which completed the case.

These models show the mouth of this patient, first, as it was before operation; secondly, as it was at five months old; thirdly, as it is at two years of age. The teeth are well erupted and show no signs of injury from the operation. The child itself shows the perfection of the results from the operation upon the lip. The upper lip is fully equal in length and width to a normal lip, with scarcely any trace of cicatricial tissue, and not noticeable by any one not looking for it.

The second patient is a young girl ten years old. She was operated on in the clinic of the school on October 26, 1900, for cleft palate. Although the lip and alveolus were perfect, her speech was very imperfect. She improved immediately upon completion of the operation, and now is ready to show you the improvement she has made up to this time.

"Where do you live?" "East Boston, Mass." "What is your name?" "Lillian." "What is your middle name?" "Phillips." "What is your last name?" "Sproule." "What is your whole name?" "Lillian Phillips Sproule." "What month is it?" "It is June." "What month were you born in?" "I was born in December."

Some of these words are among the most difficult in the English language to pronounce, yet her articulation is complete, and she is heard with perfect distinctness by every one in the room, as is proved by the response of several persons in the audience farthest from her.

DISCUSSION.

*Dr.* ———.—I would like to ask the doctor if she has had any special teaching in regard to enunciation or articulation?

*Dr. Fillebrown.*—Yes; I gave her some hints myself, and she has a school-teacher who understands what I want done and my methods of instruction, and she has taught her intelligently, and the result is her, to me, wonderful improvement. The child is very bright, and has grasped the meaning of things quickly and applied her instruction more quickly than any person I have ever met.

Pictures of these patients and of the models of the mouths were shown upon the screen and more fully described.

A paper was then read by Dr. Henry A. Kelley on "The Control of Our Patients."

(For Dr. Kelley's paper, see page 25.)

*Dr. Charles H. Taft,* Boston, Mass.—I would like to ask the doctor if he gives any preliminary talk to his patients before he begins his work?

*Dr. Kelley,* Portland, Me.—No, I do not. It seems to me the whole question is summed up in that expression, kindness of heart. The opinion expressed by Dr. Fillebrown before the National Association last August on Hypnotism pleased me very much. After repeating all the wonderful things related, he expressed his opinion and said, "I do all these things, but I use simple kindness." I remember Dr. Fillebrown replied, "I consider hypnotism is kindness of heart, and that they are one and the same thing." I contend that it is kindness of heart that gives you the control of your patients, so that when one comes in there is no need of any preparation. As soon as you begin, if you show the spirit, you have this control, for you immediately assume the responsibility, and they at once help you out. I do not give any talk about the matter. Some people have themselves under good control, but there are others whom you must control, and still others you cannot control at all.

*Dr. Charles H. Taft,* Boston.—Dr. Kelley spoke of the subject of cataphoresis, and I would like to know how many who have used it think favorably of it. I should like to hear from the gentlemen who use it. I see we have Dr. Gillett with us, and I would like to hear especially from him.

*Dr. H. W. Gillett,* Newport, R. I.—I am still using cataphoresis for certain cases, and I shall still continue to do so, for I think that it can be used satisfactorily in the majority of cases. For instance, if we have one where it is absolutely necessary to do something, where the patient demands that something be done and is willing to pay for the time it takes to use it, I think it is an excellent thing. I had, within a few days, a patient in whose family it has been used, and I prepared to fill a cavity without it, but he would not consent; he wanted me to use cataphoresis. I keep my appliances for just such patients. It is a matter of very great regret to me that others have not succeeded in it. What I hoped, when I was experimenting in helping to start you along the line of cataphoresis, was that others would be able to solve that problem, that is, the reduction of time. I do not find, myself, that I use any less time than I did then. As I am on my feet, I may, perhaps, say in answer to the question asked me, that I have yet to see any ill results from the intelligent use of cataphoresis. I question very strongly whether any ill results come from its intelligent use, but rather from its misuse.

